# Optimal Growth Conditions for *Azolla pinnata* R. Brown: Impacts of Light Intensity, Nitrogen Addition, pH Control, and Humidity

**DOI:** 10.3390/plants11081048

**Published:** 2022-04-12

**Authors:** Maria Emelia Jesus da Silva, Lebani Oarabile Joy Mathe, Ignatius Leopoldus van Rooyen, Hendrik Gideon Brink, Willie Nicol

**Affiliations:** Department of Chemical Engineering, University of Pretoria, Pretoria 0002, South Africa; dasilva.juju9@gmail.com (M.E.J.d.S.); joyoara@gmail.com (L.O.J.M.); ignatiuslvr@gmail.com (I.L.v.R.); deon.brink@up.ac.za (H.G.B.)

**Keywords:** *Azolla pinnata*, growth, pH, nitrogen, humidity, light intensity

## Abstract

Nitrogen pollution from agriculture is a major challenge facing our society today. Biological nitrogen fixation is key to combat the damage that is caused by synthetic nitrogen. *Azolla* spp. are ideal candidates for fast nitrogen fixation. This study aimed to investigate the optimal growth conditions for *Azolla pinnata* R. Brown. The growth conditions that were investigated included the growth medium type and strength, light intensity, the presence/absence of nitrogen in the medium, pH control, and humidity. Higher light intensities increased plant growth by 32%, on average. The highest humidity (90%) yielded higher growth rate values than lower humidity values (60% and 75%). The presence of nitrogen in the medium had no significant effect on the growth rate of the plants. pH control was critical under the fast growth conditions of high light intensity and high humidity, and it reduced algal growth (from visual observation). The optimal growth rate that was achieved was 0.321 day^−1^, with a doubling time of 2.16 days. This was achieved by using a 15% strength of the Hoagland solution, high light intensity (20,000 lx), nitrogen present in the medium, and pH control at 90% humidity. These optimised conditions could offer an improvement to the existing phytoremediation systems of *Azolla pinnata* and aid in the fight against synthetic nitrogen pollution.

## 1. Introduction

The impact of modern agriculture on the Earth’s natural ecosystems is severe. The advent of synthetic fertilisers propelled the green revolution, which indirectly stimulated the explosion in human population through the increased crop growth over the past half century [1]. Classical agricultural techniques had the main objective of growth efficiency, thereby causing human nutrition to become more available and affordable. Easier living has resulted in the major expansion of mankind’s footprint on our planet. Today, the consequences of this expansion are clearly visible in the alarming rate of biodiversity loss [2], global warming [3], deforestation and the availability of arable land [4], and the pollution of our waterways [5].

The nutrient pollution of Earth’s freshwater systems has become one of the main sustainability challenges that is facing mankind today. The diffusion of nitrogen and phosphorous from agricultural soils into groundwater has detrimental long-term effects on aquatic ecosystems [6]. Eutrophication occurs when excessive amounts of nutrients enter water bodies and cause algal blooms, which deoxygenate the water and cause the loss of aquatic biodiversity and poor overall water quality [7].

It is evident that novel methods for food production are urgently required in order to drastically reduce the irresponsible manner in which plant nutrients are distributed within the natural environment. On a mass basis, components that contain nitrogen are the main source of nutrition for growing food crops. Prior to the age of synthetic fertilisers, all fertilisers that contained nitrogen originated from organic sources. The nitrogen cycle entails the circulation of atmospheric nitrogen in the form of N_2_ to inorganic nitrogen in the soil and finally, organic nitrogen that is found in all living organisms [8]. Specific prokaryotes (bacteria and archaea) that contain the enzyme nitrogenase are the only organisms that are able to naturally fix atmospheric nitrogen and thus, represent the gateway for supplying nitrogen to soil and, consequently, to all living material [9].

The Harber–Bosch process, which artificially fixes atmospheric nitrogen and is used to produce synthetic nitrogen fertilisers, was first developed almost a century ago [10] and subsequently diminished mankind’s dependence on nitrogen-fixing organisms. Today, more than half of the nitrogen in global food crops originates from synthetic nitrogen [9]. However, this independence came at a significant cost: the Harber–Bosch process is highly energy intensive and requires a major amount of fossil feedstocks. It is estimated that 1.8% of global CO₂ emissions originate from this process [11]. In addition, the relatively low cost of synthetic fertiliser, as well as the increased crop productivity that is induced by the oversupply of nitrogen fertiliser, means that less than 50% of all supplied nitrogen fertiliser ends up in food crops and the rest is released into the environment [12]. The world’s burgeoning population and other environmental stressors require us to seek alternative environmentally friendly solutions for food production, water remediation, and agriculture.

Sustainable agriculture and nitrogen preservation in soil is highly dependent on a healthy soil ecosystem, in which numerous micro-organisms coexist in symbiotic relationships [13]; therefore, it is imperative to drastically reduce or even abandon the use of synthetic fertiliser in the long term. In this regard, the rise of the permaculture approach is instrumental in guiding the transition away from synthetic fertilisers and dead soil [14]. One major challenge in this initiative is the rate at which nitrogen can be fixed by prokaryotes, as crop growth is proportional to the amount of nitrogen that is available in soil. One approach to counter this challenge is to consider the centuries-old tradition of rice farmers in central Asia, whereby the aquatic fern genus *Azolla* has been used to boost nitrogen supply to rice crops. *Azolla* spp. are well known for their symbiotic relationship with the nitrogen-fixing bacteria *Anabaena azollae* [15]. *Azolla* spp. are reported to produce 70 to 110 kg nitrogen ha^−1^ [16]. Numerous studies have shown that the direct application of *Azolla* spp. to agricultural soil can dramatically reduce or eliminate the need for additional fertilisation [17].

*Azolla* spp. have been used extensively in phytoremediation processes in which the plant nutrients in water are absorbed as the *Azolla* spp. grow [18]. The growth of the *Azolla* spp. does not require any components that contain liquid phase nitrogen and accordingly, the phytoremediation process is relatively independent from the nitrogen content of the wastewater. In this regard, the *Azolla*–*Anabaena* symbiont provides an ideal solution for recycling non-nitrogen bearing nutrients from waterways back into soil. In this suggested recycling system, a major fraction of the nitrogen that is applied to soil would have originated from atmospheric nitrogen, implying that other nutrients are recycled while nitrogen is supplemented.

The biomass from the phytoremediation of *Azolla* spp. can also be applied creatively for the recovery of energy and fertiliser. The large quantities of biomass can be used as feed for anaerobic digestion, which produces biogas and digestate as fertilisers [19]. *Azolla* spp. have been called the most important macrophyte in the world due to their high biomass production rate and the fact that they are cheap and easy to grow. Potential applications for *Azolla* spp. include use in food, feed, biofuel, agriculture, and phytoremediation [20].

There have been numerous growth studies that have been performed on different species of *Azolla*. The current study focused exclusively on *Azolla pinnata* R. Brown (*A. pinnata*), which is a smaller species of the *Azolla* genus that is native to parts of coastal Africa and Asia [15]. The study aimed to carefully characterise the growth conditions for *A. pinnata* under highly controlled conditions. Various other studies have looked into the growth of *A. pinnata* and Table 1 provides some details of prominent studies.

From Table 1 it is clear that although various growth conditions have been investigated, a comprehensive comparison of the governing growth conditions is not currently available. When considering Table 1, the following parameters were identified as requiring investigation: medium type, presence of external nitrogen, effects of light (type and intensity), effects of pH, and humidity levels. In more detail:

- The variation in the media that were used in the studies complicates comparison, especially when wastewater streams were not fully characterised;
- The symbiotic relationship that all *Azolla* species have with the nitrogen-fixing bacteria *Anabaena azollae* allows the plants to live in nitrogen-free environments. However, the effects of the presence of nitrogen in the medium on growth rate have not been compared to the growth rate with an absence of nitrogen;
- Light presents many factors that must be considered in order to accurately quantify this variable. The first factor is the type of light: natural versus artificial. The second is the light intensity, which is a variable that has sometimes not been reported. The majority of the studies that are shown in Table 1 used natural light instead of artificial light. The study in [23] varied the intensity of natural light to see the effects on the growth rate of *A. pinnata*. The effects of varied artificial light conditions on growth rate have not been investigated;
- pH control as a growth condition is a clear gap in the existing literature. The pH of the medium was either adjusted initially, as in [21] and [28], or different pH values were investigated, as in [27]. There has been no comparison of the effects of non-pH-controlled versus pH-controlled conditions on the growth rate of *A. pinnata*;
- Humidity has not received serious attention, even though many studies have iterated the importance of a high humidity for the growth of the *Azolla* species [15]. In the existing *A. pinnata* studies, either the humidity was not mentioned at all or it was stated that the *A. pinnata* was grown in a greenhouse, which would increase the humidity. Only [33] and [34] reported humidity values, but these values were not controlled.

The only known growth study that attempted to assess the effects of these conditions was performed by [35], in which a simple growth chamber was built to investigate the effects of different growth conditions on *Azolla filiculoides* Lamarck and the growth of *A. pinnata*. The growth chamber investigated temperature, humidity, pH, light intensity, light colour, nutrient composition, and gas exchange. The only reported growth rate values were for *Azolla filiculoides*, with the highest growth rate reported as 0.158 day^−1^.

The current study aimed to address these gaps in the literature by utilising a controlled indoor environment to minimise the effects of extraneous factors on the results. This study investigated the optimal conditions for composition and strength, nitrogen presence, light intensity, pH control, and humidity to maximise the growth of *A. pinnata*. The growth of *A. pinnata* was compared using four different strengths of the Hoagland solution and two different strengths of the IRR2 medium [36]. The effects of three different light intensities (low light: 5000 lx, medium light: 10,000 lx, and high light: 20,000 lx) were compared. Finally, the effects of three different humidity levels (60%, 75%, and 90%) were also investigated. These conditions were investigated in combination with pH-controlled and non-pH-controlled conditions and solutions with and without nitrogen for each experimental condition. The work included filling gaps regarding the known and established growth variables. It was further hypothesised that pH control could play an important role in growth rates and that the atmospheric nitrogen fixation that occurs regardless of the presence of nitrogen in the medium would not be a rate-limiting factor due to the symbiosis between *Azolla* species and *Anabaena azollae*. The clear identification of the optimal growth conditions could guide the future development of *A. pinnata* in phytoremediation processes.

## 2. Results and Discussion

**Figure 1:**
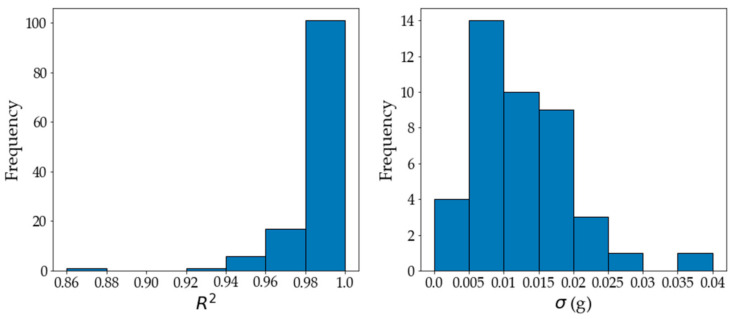
The R^2^ values of the repeats were calculated and show the measure of how closely the data fitted the exponential line of regression, represented by Equation (1), with the corresponding growth rates. The standard deviations were calculated for the repeats of each experiment by comparing the growth rate of the repeats against the average growth rate value of that set.

The starting mass of *A. pinnata* (0.25 g) was placed into 1 L cylindrical containers that contained a specified medium. Every day, the medium’s pH was measured and adjusted with acid/base dosing to maintain a pH of 6.5 for the pH-controlled experiments. In triplicate repeats, the containers were each placed under LED lightbulbs with a specified light intensity. The setup was located inside a walk-in greenhouse with a controlled humidity. The plant masses and photographs were recorded on days 0, 1, 3, 5, and 7 of the 7-day run. A total of 42 experiments were conducted, which were all performed in triplicate. The repeatability of the triplicate experiments was generally good. Figure 1 shows the coefficients of determination (R^2^) and standard deviation (σ) values of the repeat data.

The growth rate for each repeat was determined using the scipy.optimize.curve_fit module. The R^2^ values were calculated for each triplicate to determine the quality of the fit in correspondence to the determined function. The worst fit resulted in an R^2^ value of 0.870. This was clearly an outlier, since 99.20% of the R^2^ values were above 0.9 and 96.03% of the values were above 0.95. An average growth rate, μ (day^−1^), for each experiment was calculated. The standard deviations were calculated using the growth rate for each repeat compared to the average growth of the set for one experiment. The standard deviations were low (maximum σ < 0.04 compared to average growth rates of 0.123–0.192 day^−1^), indicating that the growth rates of the repeats tended to be close to the average growth rate of the set. Consequently, only the average values for the experimental graphs were reported.

**Figure 2:**
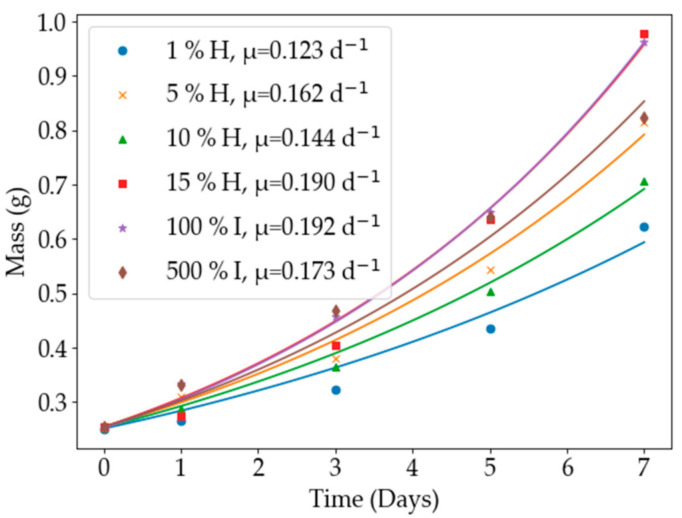
The average weight values are shown for each of the growth mediums, the Hoagland solution (H) and the IRR2 medium (I), along with the optimised growth curve and growth rate. The 15% strength Hoagland solution and the 100% strength IRR2 medium produced optimal growth.

In the first experiment, the effect of medium composition was investigated in order to determine the optimum medium for use in the remainder of the study. The six media that were investigated were 1%, 5%, 10%, and 15% strengths of the Hoagland solution and 100% and 500% strengths of the IRR2 medium. A detailed description of the media can be found in Section 3.1. The light intensity was set to medium light (10,000 lx) and the humidity was 75%. All of the media contained nitrogen and pH control was not implemented. Figure 2 shows the average mass of the triplicates and the optimised curve with the corresponding growth rates.

**Figure 3:**
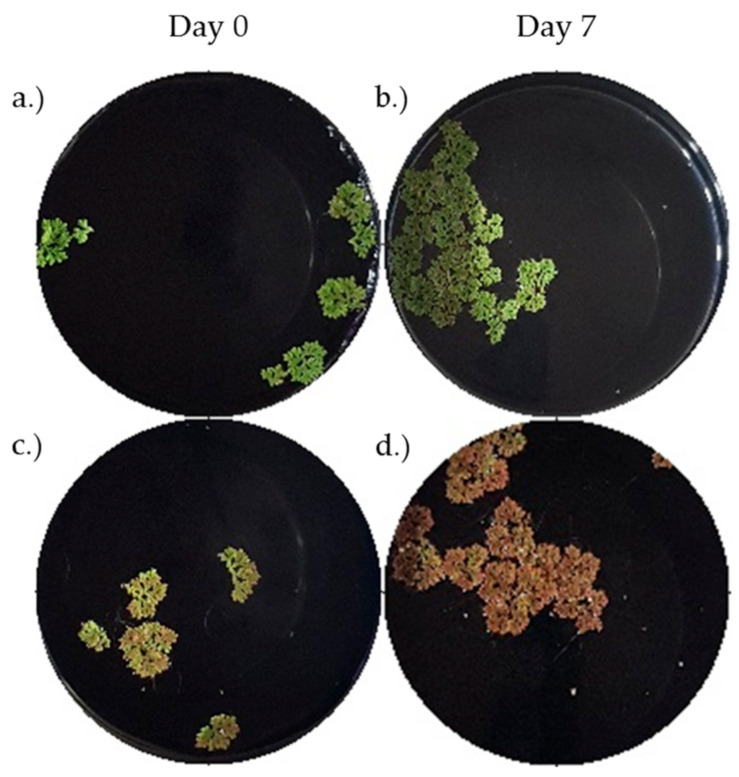
Photographs of the *A. pinnata* were taken throughout the experiment. These images demonstrate a comparison of the 15% strength Hoagland solution (image (**a**,**b**)) and the 100% strength IRR2 medium (image (**c**,**d**)). All other growth conditions were constant: nitrogen was present, no pH control, medium light intensity (10,000 lx), and 75% humidity. It was concluded that the Hoagland solution was better suited to healthy plant growth due to the consistent green colour compared to the red-brown colour produced by the IRR2 growth medium, which indicated that the plant was stressed.

It can be noted that the 100% IRR2 medium achieved the highest growth rate of 0.192 day^−1^. The 15% Hoagland solution achieved a comparable growth rate of 0.190 day^−1^. This contrasted to the research of [33], in which the optimal growth rate of 0.124 day^−1^ was achieved using a full strength Hoagland solution. The lowest growth rate was 0.123 day^−1^, which was achieved using the 1% Hoagland solution. This was probably due to the extremely low amount of nutrients that were available in contrast to the higher strength mediums. The colour of the *Azolla* that were grown in the 100% IRR2 medium was a dark red, as shown in Figure 3. The presence of anthocyanin pigments shows that the plants were under stress, usually because of nutrient deficiency or high light intensity [37]. The plants that were grown in the Hoagland solutions remained a healthy green colour. Consequently, the 15% Hoagland solution was selected as the growth medium for the rest of the experiments based on the visual health of the plants and the observation that the maximum growth rates for the different media were practically the same.

**Figure 4:**
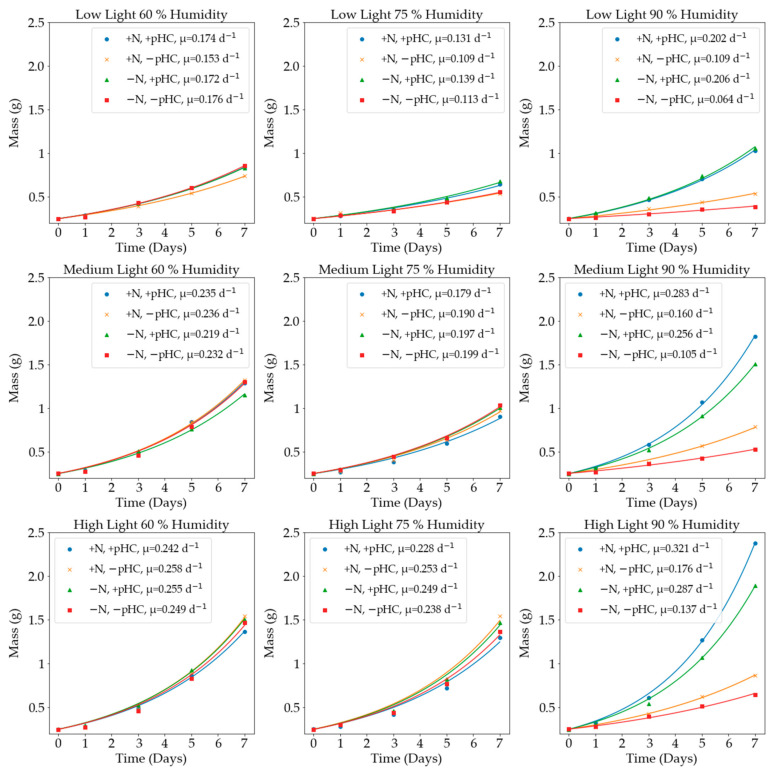
A comparison between the experiments for the presence (+N) versus absence (−N) of nitrogen, pH control (+pHC) versus non-pH control (−pHC), different light intensities (low: 5000 lx, medium: 10,000 lx, and high: 20,000 lx), and different humidity values (60%, 75%, and 90%). The average weight values are plotted, along with the optimised growth curve and the growth rate.

Figure 4 compares the growth rates for the following growth conditions: light intensity, the presence or absence of nitrogen, pH control, and humidity. In addition, the results from the one-way ANOVA analysis for these conditions are summarised in Supplementary Figure S1.

From the results, it can be seen that light intensity had a strong correlation to the growth rate. The higher light intensities caused the *A. pinnata* to produce higher final masses and therefore, higher growth rates. Previous studies with varied natural light of 30,000 lx to 120,000 lx [21], 15,000 lx to 80,000 lx [23], and 40,500 lx to 64,800 lx [26] reported significantly reduced average growth rates of 0.126 day^−1^, 0.085 day^−1^, and 0.056 day^−1^, respectively. In some conditions, the higher light intensity caused the plants to turn red. The red colour was noticed at most humidity values when nitrogen was not present, regardless of whether there was pH control. The lack of the nitrogen coupled with a high light intensity was the probable cause for the presence of the red colour, which indicated the plant was under environmental stress. From low to medium light intensity, there was an average of a 46% increase in mass and from medium to high light intensity, there was an average of a 17% increase in mass. On a light intensity basis, the largest growth difference was obtained between low and medium light intensities for the natural, no nitrogen run at 75% humidity. The light intensity caused a 76% increase in mass. It can be seen in Figure 4, which compares the humidity values and changing light intensities, that there was a clear trend that linked light intensity and growth rate. 

Figure 4 and Figure S1 show that the presence of nitrogen did not significantly affect *A. pinnata* growth. Each of the subplots in Figure 4 include the presence and absence of nitrogen counterparts for comparison. The largest difference between the growth rate values was 0.055 day^−1^ for the 90% humidity run with medium light and no pH control. The average increase in growth for the presence of nitrogen in the medium compared to the nitrogen-free medium was 6.3%. This marginal number suggests that the energy requirements for nitrogen fixation was not a rate-limiting factor for growth under the conditions that were employed. The small differences were most likely linked to the initial conditions in which the *Anabaena azollae* population adapted to the nitrogen-free liquid, since the preparatory growth prior to the experiment was performed in a nitrogen-rich medium. This result clearly exhibits the nitrogen fixing potential of *A. pinnata* and shows that other growth factors determined the extent of nitrogen fixation. Previous studies did not conduct the requisite comparison between the presence and absence of nitrogen in the medium; therefore, the previous studies are not comparable to this study.

**Figure 5:**
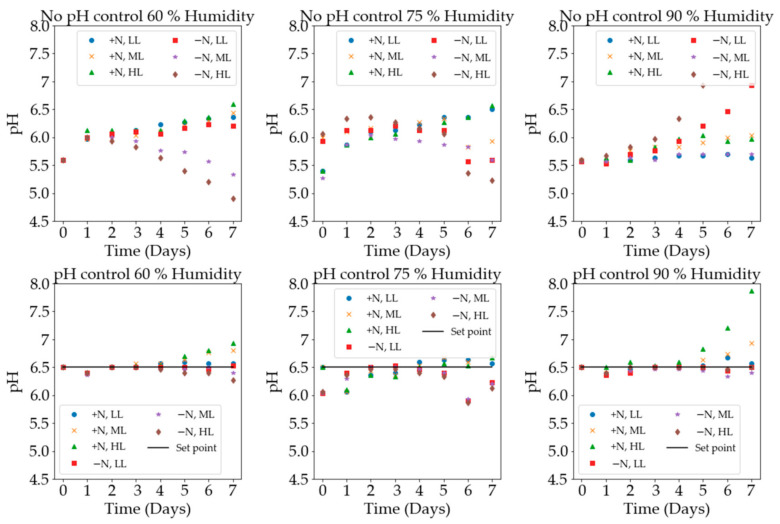
The pH data were measured every day before acid/base dosing. The different light intensities (LL: 5000 lx, ML: 10,000 lx, and HL: 20,000 lx) and the nitrogen presence (+N) or absence (−N) are also shown. The set-point value of 6.5 was selected for the pH-controlled experiments.

pH-control was performed via the daily dosing of acid or base to maintain the pH at the set-point value of 6.5. The uncontrolled experiments were not dosed at all and had a starting pH of between 5.5 and 6. Previous studies set initial pH values, however, did not control the pH thereafter [27]. One study did control the pH at a value of 6.1 [28], but there were no uncontrolled data for comparison. The growth rate was 0.300 day^−1^, indicating that pH control could be a significant factor in optimising growth. The experiments in which the growth medium contained nitrogen and the pH was not controlled showed a general trend of increasing pH. In the nitrogen-free mediums without pH control, the pH usually decreased. Figure 5 shows the daily pH data for the controlled and uncontrolled experiments under the respective growth conditions. 

The exception to these trends was the experiment with high light intensity, without nitrogen, and 90% humidity. All of the containers (the repeats) became infected with thick green algae and reached the extremely high pH values of 9.6, 9.2, and 9.6. This was an average pH of 9.5. This high pH was thought to have been caused by the algal infection that was clearly visible in the containers. Without the pH being controlled and in a high light intensity and high humidity environment, the conditions were perfect for algae infection. This negatively affected the growth of the plants, as they only achieved a growth rate of 0.176 day^−1^. The counterpart pH-controlled experiment achieved a growth rate of 0.321 day^−1^. There was some algae growth present and the final pH value, before dosing, was 7.9; however, the growth was evidently much higher and the visual health of the plant was much better. This difference in growth rate that was solely due to the pH control can be observed in Figure 4, in the high light intensity and 90% humidity subplot.

The high light intensity experiments were conducted under the same conditions, except for varying humidity values. The 60% and 75% humidity values produced similar results overall, but the 60% humidity experiments achieved slightly higher growth rates. The increase in growth rate for the 60% humidity compared to the 75% humidity was 5.8%, 2.4%, 1.2%, and 4.4%. There were two growth studies that reported humidity values: one reported 60% to 70% [33] humidity and the second reported 55% to 70% humidity [34]. Both studies reported the lower growth rates of 0.124 day^−1^ and 0.100 day^−1^, respectively. The 90% humidity had a significant effect. The rate of transpiration increased at higher humidity values, which promoted higher growth values but there was an increased chance of mould and algae that could negatively affect the growth. The pH-controlled runs increased growth by 24% and 11%. There was a 32% and 45% reduction in growth for the non-pH-controlled runs. This was due to the algal infections that severely impacted the growth of the *A. pinnata*.

## 3. Materials and Methods

### 3.1. Materials

The *A. pinnata* was collected from the Manie van der Schijff Botanical Gardens at the University of Pretoria. It was grown in a deep pond in a misted greenhouse. 

This study opted for two well-formulated synthetic media to standardise the experimental conditions for the presence and absence of nitrogen. The following liquid media were prepared as growth media. The Hoagland solution (per litre) comprised 0.120 mg of Cu–EDTA–2Na, 0.240 mg of ZnSO_4_•7H_2_O, 1.80 mg of MnCl_2_•4H_2_O, 0.490 g of MgSO_4_•7H_2_O, 0.0190 g of Fe–EDTA, 0.740 g of CaCl_2_•H_2_O, 2.88 mg of H_3_BO_3_, 0.120 mg of Na_2_MoO_4_•H_2_O, and 0.136 g of KH_2_PO_4_ (plus 0.505 g of KNO_3_ for the medium that contained nitrogen).

One litre of the IRR2 solution contained 69.7 mg of K_2_SO_4_, 98.60 mg of MgSO_4_•7H_2_O, 1.90 mg of Fe–EDTA, 58.8 mg of CaCl_2_•H_2_O, 13.6 mg of KH_2_PO_4_, 25.0 μg of CuSO_4_•5H_2_O, 0.120 mg of H_3_BO_3_, 36.0 mg of Na_2_MoO_4_•2H_2_O, 29.0 μg of ZnSO_4_•7H_2_O, 24.0 μg of CoCl_2_•6H_2_O, 99.0 mg of MnCl_2_•4H_2_O, and 1.4.0 mg of FeSO_4_•7H_2_O.

The pH was adjusted by adding 0.250 M of NaOH or 0.100 M of HCl as required.

### 3.2. Analytical Instruments

The wet mass of the plants was measured using an OHUAS™ Adventurer 2 digital lab scale. The light intensity was measured using a handheld digital Victor A1010 lx meter. The pH was measured using a digital Bluelab™ combo pH and EC meter, Bluelab, Tauranga, New Zealand. The humidity was measured using a digital temperature and humidity sensor (SHT10), Sensirion, Stäfa, Switzerland, coupled with an Arduino MEGA 2560™, Smart Projects, Ivrea, Italy.

### 3.3. Experimental Procedure

#### 3.3.1. Structure

A 143 × 73 × 195 cm walk-in greenhouse was constructed with a total of six shelves. On each shelf were three 1 L containers with a diameter of 0.115 m and a height of 0.1 m, which were used as the repeats for the experiment. The *A. pinnata* was collected from the greenhouse and washed in deionised water. The plants were then dried on paper towels to remove excess water. A total of 0.25 g of *A. pinnata* was weighed out as the starting mass.

#### 3.3.2. Growth Media

The desired solutions and strengths were prepared by adding nitrogen, depending on the experiment. The solution was diluted with deionised water to reflect the desired strength on a mass basis. Low concentrations were chosen due to the small starting mass of *A. pinnata* and the low nutrient requirements of the plants, according to the literature. The containers were each filled with a specific solution and the starting mass of *A. pinnata.*

#### 3.3.3. pH Control

The pH of the solution was measured daily. For the pH-controlled runs, daily dosing of the or base was administered to the container to keep the pH constant at 6.5. The uncontrolled experiments were not dosed and the initial pH value varied between 5.5 and 6.

#### 3.3.4. Lighting

The 1 L containers containing the desired solution and *A. pinnata* with the controlled or uncontrolled pH levels were each placed under a light bulb. The lights that were used were custom constructed for this experimental setup. Three 9 W, Eurolux light bulbs with an E27 base were used. The bulbs were cool white, dimmable, LED globes. These globes were attached to ceramic E27 base fittings. Each fitting was wired to a 1 m long cabtyre 3-core 1.5 mm wire, which was soldered in parallel to a 2.5 m wire of the same dimensions. A total of six light setups were made. The plugs of the wired globes were plugged into a Major Tech -MTD3 programmable 24 h timer. This was to allow for a day/night cycle of 16 h/8 h. The plants were grown in 1 L cylindrical, black PVC containers to reduce algae growth. The light intensity was set using the lux meter and a combination of the height of the bulb and the rotary switch to dim or brighten the lights to the desired intensity. Three different light intensities were used: low light (5000 lx), medium light (10,000 lx), and high light (20,000 lx). This range was chosen due to the physical constraints of the custom light design. The increments were chosen due to the significant differences in light intensity.

#### 3.3.5. Humidity

**Figure 6:**
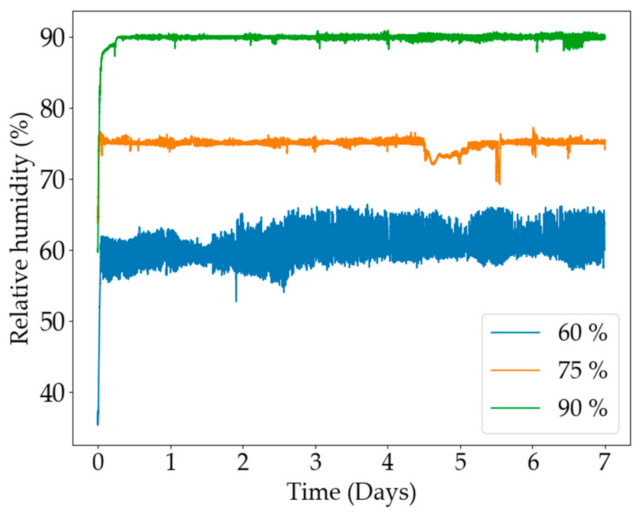
The on/off humidity control data for the three different humidity set-points.

An Elektra Health 5 L humidifier was used to regulate the humidity in the greenhouse. An Arduino MEGA 2560™ was coupled with a humidity sensor and the humidifier to employ a simple on/off control scheme to achieve the desired set-point. A set-point for the humidity in the greenhouse was decided and the humidifier was switched on/off to maintain the designated set-point and the humidity values were recorded. The humidity controls at three different set-points are shown in Figure 6. The 60% humidity set-point had an average humidity value of 60.81% and a standard deviation of 2.58%. The 75% humidity set-point had an average value of 74.99% and a standard deviation of 0.77%. The 90% humidity set-point value had an average value of 89.83% and a standard deviation of 1.29%. Overall, humidity control was considered very effective. 

#### 3.3.6. Measurements and Growth Analysis

The experiment was run for 7 days. The plants were photographed and removed from the medium, dried on paper towel to remove excess water, and weighed on days 0, 1, 3, 5, and 7. The masses were recorded.

The mass values that were measured over the run of the three repeats were averaged to obtain five average mass points over a 7-day period. The scipy.optimize.curve_fit module in Python™ was used to find the optimal set of parameters for a defined function that minimise the error of a set of data points. An exponential curve, shown as Equation (1), was set as the defined function to find the growth rate, *μ* (day^−1^).

*M_f_* = *M_i_ e ^μ t^* (1)

where *M_f_* (g) and *M_i_* (g) are the final and initial mass values, respectively, and *t* (days) is time.

To test the significance of the differences between the experimental conditions, a one-way ANOVA test was performed using the biomass measurements from the first day and the final day of the experimental runs. This was conducted to determine whether a significant difference between runs at the beginning and end of each experiment could be observed. The analysis was performed using the Graphpad Prism 9 software package (GraphPad Software Inc., San Diego, CA, USA).

## 4. Conclusions

This growth study of *A. pinnata* investigated solution type and strength, light intensity, nitrogen presence, and humidity values. Using the 15% Hoagland solution, the following was concluded. The growth conditions that resulted in the slowest growth rate of 0.064 day^−1^ were low light intensity, 90% humidity, no nitrogen, and no pH control. The growth conditions that achieved the highest growth rate of 0.321 day^−1^ were high light intensity, 90% humidity, nitrogen, and pH control. A higher light intensity resulted in higher growth rates. The presence or absence of nitrogen generally produced similar growth rates. The pH control had the greatest effect at high humidity and reduced algae formation, thus improving the health of the plants and increasing the growth rate. Overall, the study concluded that higher humidity values increased the plant growth rate but needed to be used in conjunction with pH control. High biomass yields could be obtained by utilising the conditions that were found to promote optimal growth. This biomass could then be used for phytoremediation processes, along with other applications.

## Figures and Tables

**Table 1:** A literature comparison of studies that recorded the growth of *A. pinnata* under different growth conditions. A summary of the aims of each paper is provided. The growth medium, pH control, presence of nitrogen, light intensity, and humidity control are all noted. The average specific mass-based growth rate *μ* (day^−1^) that was reported in each study is also provided.

Summary	Growth Medium	pH Control	Nitrogen Presence/Absence	Light Intensity Comparison ^1^	Humidity Control	*μ* (Day^−1^)	Reference
*A. pinnata* growth and nitrogen fixation symbiosis	Fertiliser and floodwater	Not controlled	Present	Varied natural light; 30,000 lx to 120,000 lx	Not measured	0.126	[21]
*A. pinnata* influence on rice crops	Fertiliser and floodwater	Not controlled	Present	Natural light; not quantified	Greenhouse conditions	0.110	[22]
Effects of light intensity on *A. pinnata*	Fertiliser with floodwater	Not controlled	Present	Varied natural light; 15,000 lx to 80,000 lx	Not measured	0.085	[23]
Effects of photoperiods on the growth of *A. pinnata*	Fertiliser with tap water	Not controlled	Present	Artificial light; 7500 lx	Not measured	0.090	[24]
Water remediation of sewage using *A. pinnata*	Sewage and tap water	Not controlled	Present	Natural light; not quantified	Greenhouse conditions	0.025	[25]
Effects of light intensity on *A. pinnata* grown in effluent	Sewage and tap water	Not controlled	Present	Varied natural light; 64,800 lx to 40,500 lx	Greenhouse conditions	0.056	[26]
Growth of *A. pinnata* under different growth conditions	Nutrient enriched water	Different initial pH values investigated; not controlled	Varied presence	Varied natural light; not quantified	Greenhouse conditions	0.283	[27]
Growth comparison of *A. pinnata* strains	Standard medium [16]	pH controlled at 6.1; no uncontrolled data	Present	Artificial light; 10,800 lx	Not measured	0.300	[28]
Effects of varying salinity on *A. pinnata* growth	Hoagland solution with 10 mM of NaCl added	Not controlled	Absent	Artificial light; 5130 lx	Not measured	0.037	[29]
Water remediation of sewage using *A. pinnata*	Sewage and tap water	Not controlled	Present	Natural light; not quantified	Not measured	0.251	[30]
Growth comparison of *A. pinnata* in the presence of pesticides	Standard medium [16] with pesticide	Not controlled	Present	Artificial light; 10,800 lx	Not measured	0.0650	[31]
Chemical composition of sun-dried *A. pinnata*	Fertiliser with floodwater	Not controlled	Present	Natural light; not quantified	Not measured	0.139	[32]
Growth analysis of *A. pinnata* in greenhouse conditions	Hoagland solution	Not controlled	Absent	Natural light; not quantified	Greenhouse conditions; 60% to 70%	0.124	[33]
Phytoremediation of dairy wastewater using *A. pinnata*	Dairy wastewater	Not controlled	Present	Artificial light; 2000 lx	Laboratory conditions; 55% to 70%	0.100	[34]

^1^ Units of lx indicate the SI derived light intensity units if of lux (luminous flux per square meter).

## Data Availability

The data presented in this study are openly available from the University of Pretoria Research Data Repository at doi:10.25403/UPresearchdata.19550797.

## Supplementary Materials

The following supporting information can be downloaded at: https://www.mdpi.com/article/10.3390/plants11081048/s1, Figure S1: A heat map showing the adjusted *p* values when comparing the repeated results from the individual experimental conditions. The colour scale shows comparisons with no significant differences (white) to most significant differences (dark green).
