# Supplementary material for: Optimal Growth Conditions for Azolla pinnata R. Brown: Impacts of Light Intensity, Nitrogen Addition, pH Control, and Humidity
Source: Plants (Basel). 2022 Apr 12;11(8):1048. doi: 10.3390/plants11081048 (PMC9032100; doi:10.3390/plants11081048)
Supplement: Supplementary file 1 [file plants-11-01048-s001.zip › plants-1653693-supplementary.pdf]

Supporting Information

Optimal Growth Conditions of Azolla pinnata

Maria Emelia Jesus da Silva, Lebani Oarabile Joy Mathe, Ignatius Leopoldus van Rooyen, Hendrik Gideon Brink and Willie Nicol *

Department of Chemical Engineering, University of Pretoria, Pretoria 0002, South Africa; dasilva.juju9@gmail.com (M.E.J.d.S.); joyoara@gmail.com (L.O.J.M.); ignatiuslvr@gmail.com (I.L.v.R.); deon.brink@up.ac.za (H.G.B.)

* Correspondence: willie.nicol@up.ac.za

S1 One-way ANOVA test for significant difference

A one-way ANOVA analyses performed between the triplicate biomass measurements taken on day one and seven for the experimental conditions tested. The results from day 1 showed that no significant difference could be observed between any of the runs. The results for the day 7 biomass measurements are shown in the heat map (Figure S1). The colours are scaled according to the significance level with white representing an insignificant difference and dark green showing a very significant difference in the measurements.

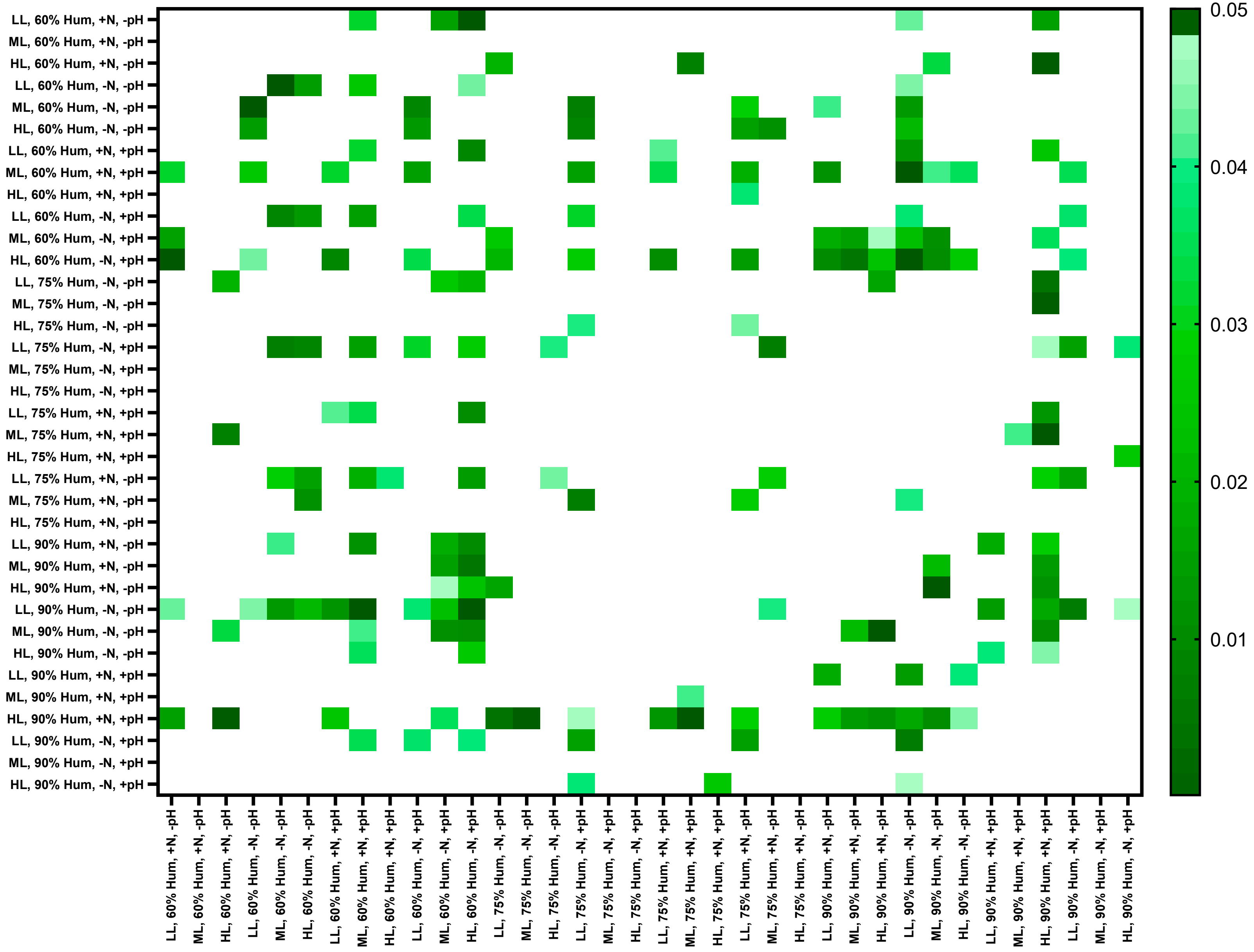

Figure S1: A heat map showing the adjusted P values when comparing the repeated results from the individual experimental conditions. The colour scale shows comparisons with no significant differences (white) to most significant differences (dark green).
